# Ecto-Phosphorylation of CD98 Regulates Cell-Cell Interactions

**DOI:** 10.1371/journal.pone.0003895

**Published:** 2008-12-09

**Authors:** Hang Thi Thu Nguyen, Guillaume Dalmasso, Yutao Yan, Tracy S. Obertone, Shanthi V. Sitaraman, Didier Merlin

**Affiliations:** Division of Digestive Diseases, Department of Medicine, Emory University School of Medicine, Atlanta, Georgia, United States of America; University of Washington, United States of America

## Abstract

Ecto-phosphorylation plays an important role in many cellular functions. The transmembrane glycoprotein CD98 contains potential phosphorylation sites in its extracellular C-terminal tail. We hypothesized that extracellular signaling through ecto-protein kinases (ePKs) might lead to ecto-phosphorylation of CD98 and influence its multiple functions, including its role in cell-cell interactions. Our results show that recombinant CD98 was phosphorylated *in vitro* by ePKs from Jurkat cells and by the commercial casein kinase 2 (CK2). Alanine substitutions at serines-305/307/309 or serines-426/430 attenuated CK2-mediated CD98 phosphorylation, suggesting that these residues are the dominant phosphorylation sites for CK2. Furthermore, CD98 expressed in the basolateral membranes of intestinal epithelial Caco2-BBE cells was ecto-phosphorylated by Jurkat cell-derived ePKs and ecto-CK2 was involved in this process. Importantly, cell attachment studies showed that the ecto-phosphorylation of CD98 enhanced heterotypic cell-cell interactions and that the extracellular domain of CD98, which possesses the serine phosphorylation sites, was crucial for this effect. In addition, phosphorylation of recombinant CD98 increased its interactions with Jurkat and Caco2-BBE cells, and promoted cell attachment and spreading. In conclusion, here we demonstrated the ecto-phosphorylation of CD98 by ePKs and its functional importance in cell-cell interactions. Our findings reveal a novel mechanism involved in regulating the multiple functions of CD98 and raise CD98 as a promising target for therapeutic modulations of cell-cell interactions.

## Introduction

CD98, a type II transmembrane glycoprotein, is a potential regulator of multiple functions, including extracellular signaling, epithelial cell adhesion/polarity, amino-acid transport and cell-cell interactions [1]. Following its discovery as a surface antigen in lymphocytes [2], CD98 has been found to be expressed in all cell types with the exception of platelets, and is expressed at highest levels in the gastrointestinal tract and the tubules of the kidney [1]. In intestinal epithelial cells (IECs), CD98 is targeted to the basolateral membranes and forms heterodimers with amino-acid transporters. These CD98/amino-acid transporter heterodimers have been shown to associate with β_1_-integrin and function as a β_1_-integrin regulator [3], [4], [5], [6].

Extracellular (Ecto-) phosphorylation is emerging as an important mechanism in the regulation of many physiological processes, such as cell-cell interactions, cellular differentiation and proliferation, ion fluxes and cellular activation [7]. A variety of cells, such as immune cells, have been reported to possess ecto-protein kinases (ePKs) [7]. ePKs have been identified as plasma membrane-associated protein kinases that act on the outer surface of cells [8]. ePKs, which can be released from intact cells in a process termed “shedding” [9], are capable of phosphorylating cell-surface proteins, extracellular matrix proteins and soluble substrates using extracellular ATP as a phosphate donor. Casein kinase 2 (CK2) is a highly conserved ePK expressed in nearly every eukaryotic tissue and cellular compartment [10], [11], and can phosphorylate numerous proteins [12], [13]. Although CK2 does exhibit some tyrosine kinase activity, it mainly phosphorylates serine or threonine residues [10].

Inspection of the human CD98 primary sequence revealed the presence of potential phosphorylation sites in its C-terminal extracellular domain. We therefore hypothesized that extracellular signaling through ePKs might lead to ecto-phosphorylation of CD98 and influence its multiple functions. Since cell-cell interactions are of great importance in the functioning of the immune system, we searched for a phosphorylation of CD98 by ePKs from T lymphocytes and further examined whether the ecto-phosphorylation of CD98 could modulate heterotypic interactions between IECs and T lymphocytes.

## Materials and Methods

### Cell culture

The human intestinal epithelial Caco2-BBE cell line and CHO cells were grown in DMEM (Invitrogen, Carlsbad, CA) supplemented with 10% FBS (Invitrogen) and 1.5 µg/ml plasmocin (Invivogen). The human T-lymphoblastoid Jurkat E6-1 cell line was cultured in RPMI 1640 (Invitrogen) supplemented with 10% FBS and 1.5 µg/ml plasmocin.

### Plasmid construction and transfection

The CD98/pTarget plasmid, constructed as previously described [5], was used as a template to generate CD98 construct variants. Full-length CD69 was cloned from macrophage KG1 cell line. CD69-CD98 chimeras were constructed by PCR-mediated overlap extension. CD69 truncated mutant lacking the extracellular domain (amino acid 62–199*) was generated using specific primers p3-ATG AGC TCT GAA AAT TGT TTC GTA GC and p4-GAA GAG CAG CAG CAG TGC CCA TTG GAA GGA CCC TTC ATC. CD98 truncated mutant lacking the cytoplasmic and transmembrane domains (amino acids 1–104*) was generated using specific primers p1-CAT GAA GGG TCC TTC CAA TGG GCA CTG CTG CTG CTC TTC and p2-GGC CGC GTA GGG GAA GCG GAG CAG CAG. PCR products were added together to a further PCR using Platinum DNA polymerase (Invitrogen) and specific primers p3 and p2 to obtain C69-T69-E98 sequence. Wild-type CD98 and the chimeras were subcloned into the pcDNA3.1/V5-His-TOPO vector (Invitrogen). *Numbering was based on the amino acid sequence reported for human CD69 (entry Q07108) and human CD98 (4F2; entry P08195) of the Swiss-Prot database.

The CD98-pcDNA3.1/V5-His-TOPO S305A-S307A-S309A mutant was generated by changing the codon at 1440–1442*, 1446–1448* and 1452–1454* position from serine 305, 307 and 309, respectively, to alanines using specific primers P1 (forward, 5′-C TCA TAC CTG GCT GAT GCT GGC GCC ACT GGG GAG C-3′; reverse, 5′-G CTC CCC AGT GGC GCC AGC ATC AGC CAG GTA TGA G-3′). The CD98-pcDNA3.1/V5-His-TOPO S426A-S430A mutant was generated by changing the codon at 1803–1805* and 1815–1817* position from serine 426 and 430, respectively, to alanines using specific primers P2 (forward, 5′-TTC CGG CGG CTA GCT GAC CAG CGG GCT AAG GAG CGC-3′; reverse, 5′-GCG CTC CTT AGC CCG CTG GTC AGC TAG CCG CCG GAA-3′). Site-directed mutagenesis by PCR-mediated overlap extension was performed using QuikChange™ Site-Directed Mutagenesis Kit (Stratagene, La Jolla, CA). *Based on NCBI NM002394 numbering; 1^st^ methionine (start of translation) = 225.

CHO cells were transfected with the constructs using Lipofectin (Invitrogen) and stably selected in culture media supplemented with 1.2 mg/ml geneticin (Invitrogen).

### Generation of ***Tet***R Caco2-BBE cell line containing CD98-shRNA constructs

Caco2-BBE cells were first transfected with the pcDNA4/TO/myc-His/lacZ vector (Invitrogen), which encodes the tetracycline repressor (*Tet*R). This *Tet*R Caco2-BBE cell line was stably selected in DMEM supplemented with 10% Tet system-FBS (Clontech) and 10 µg/ml blasticidin (Invivogen).

Three CD98-siRNA candidates were purchased from Genscript (siRNA1: J02939-680*-76 bp; siRNA2: J02939-972*-76 bp; siRNA3: J02939-1130*-76 bp. *Positions in the hCD98 sequence, accession no. NCBI NM_002394). Short hairpin (sh) constructs were designed, generated and introduced into pRNATin-H1.2/Hygro vector by GenScript. The *Tet*R Caco2-BBE cell line was transfected with the pRNATin-H1.2/Hygro empty vector or CD98-shRNA constructs and stably selected in DMEM supplemented with 10% Tet system-FBS, 10 µg/ml blasticidin and 500 µg/ml hygromycin (Invivogen).

### Recombinant protein production

The CD98-pRSET/His-B plasmid was constructed as previously described [14]. CD98-pRSET/His-B S305A-S307A-S309A and CD98-pRSET/His-B S426A-S430A mutants were generated using specific primers P1 and P2 as described for the generation of hCD98-pcDNA3.1/V5-His-TOPO S305A-S307A-S309A and hCD98-pcDNA3.1/V5-His-TOPO S426A-S430A, respectively. These vectors were transformed into *E. coli* strain BL21(DE3) pLys (Novagen, Madison, WI), grown in LB media and induced with 0.1 M IPTG for 3 h. The cells were lysed with BugBuster Protein Extraction Reagent (Novagen), sonicated and centrifuged. The insoluble pellet was solubilized in 6 M urea for 1 h and centrifuged again. CD98 was purified from the urea soluble fraction by elution off a HisBind Resin column following the protocol of the HisBind Purification kit (Novagen) with the following modifications: buffers were prepared with the addition of 6 M urea. Column was washed with 4 vol of binding buffer, 4 vol of 10 mM imidizole washing buffer and 4 vol of 15 mM imidizole washing buffer. CD98 was eluted with 3 vol of 120 mM imidizole elution buffer and 3 vol of 240 mM imidizole elution buffer. 5 µl samples from each fraction were run on 10% polyacrylamide gel and either stained with Coomassie Blue or transferred to a nitrocellulose membrane for Western blot analysis.

### Preparation of ePKs from Jurkat cells

Intact Jurkat cells were incubated with dephosphorylated α-casein (Sigma, St Louis, MO) and the released ePKs were purified as previously described [15]. Briefly, Jurkat cells adhered to a 96-well plate (5×10^5^ cells/50 µl/well) were incubated with 250 µg/ml dephosphorylated α-casein for 10 min at 37°C. The supernatants were pooled, and 10 mM diisopropylfluorophosphate was added. The sample was centrifuged at 14,000 *g* for 10 min at 4°C. The resulting supernatant was finally centrifuged at 100,000 *g* for 1 h at 4°C and stored at −70°C until use.

### *In vitro* kinase assay

Recombinant CD98 (1 µg) was incubated with 50 ng CK2 (Upstate, Dundee, UK) in a buffer containing 30 mM Tris-HCl pH 7.6, 1 mM EGTA, 150 mM NaCl, 50 mM KCl, 10 mM MgCl_2_, 40 µM ATP, 2 µCi [γ-^32^P]ATP (30 Ci/mmol, Amersham, Piscataway, NJ), 100 µM Na_3_VO_4_, 100 µM NaF and 0.05% Triton X-100, in a total volume of 25 µl, at 30°C for 10 min. The reaction was terminated by addition of Laemmli sample buffer, and samples were heated at 100°C for 5 min and loaded on to 10% acrylamide gels. Gels were stained with Coomassie Blue, destained and dried. Radiolabeled bands were detected and quantified on a Typhoon 9200 PhosphorImager (Amersham Pharmacia Biotech). Kinetics of the CK2-mediated CD98 phosphorylation were determined by performing *in vitro* kinase assays with different concentrations of recombinant CD98 and constant amounts of CK2 (50 ng) and [γ-^32^P]ATP (2 µCi). Kinetic constants were determined from a Michaelis-Menten plot using Enzyme Kinetic Program.

Phosphorylation of recombinant CD98 by ePKs from Jurkat cells was performed by mixing 40 µl ePKs with 20 µ1 NaCl-HEPES for 10 min at 23°C. 20 µl NaCl-HEPES containing 10 µCi [γ-^32^P]ATP, 1 µg recombinant CD98, 400 µM MnC1_2_, 800 µM Na_3_VO_4_ and 800 µM NaF was added. The reaction was performed at 37°C for 10 min. Samples were analyzed by SDS-PAGE. Radiolabeled bands were detected by autoradiography. Control reactions were performed under the same condition in the absence of CD98, [γ-^32^P]ATP or CK2/ePKs, or in the presence of myelin basic protein (Upstate, Lake Placid, NY). Inhibition kinase assay was performed in the presence of 10 µM of the broad-spectrum ePK inhibitor K252b (Calbiochem).

### Ecto-protein kinase assay

Ecto-phosphorylation of CD98 expressed in Caco2-BBE cells was performed using Jurkat cell-derived ePKs. 400 µ1 ePKs was mixed with 200 µ1 NaCl-HEPES and incubated for 10 min at 23°C. 200 µl NaCl-HEPES containing 100 µCi [γ-^32^P]ATP, 400 µM MnC1_2_, 800 µM Na_3_VO_4_ and 800 µM NaF was added. The reaction mixture was added into the basolateral compartment of Caco2-BBE monolayers grown on filters for 10 min at 37°C. Inhibition kinase assays were performed in the presence of 10 µM of the ePK inhibitor K252b or 10 µM of the CK2-specific inhibitor 4,5,6,7-Tetrabromobenzotriazole (TBB, Calbiochem). Cells were washed with PBS and lysed in RIPA buffer as previously described [16]. Cell lysates were immunoprecipitated for CD98 or the irrelevant IgG_1_ using Catch and Release Reversible Immunoprecipitation kit (Upstate Biotech).

Ecto-phosphorylation of CD98 in CHO cells by intact Jurkat cells was performed as previously described [17] with some modifications. Briefly, CHO cells in 12-well plates and suspended Jurkat cells were washed with ecto-kinase buffer (HBSS, 10 mM HEPES pH 7.4, 10 mM glucose, 10 mM MgCl_2_). The reaction was initiated by adding 10^7^ Jurkat cells in 400 µl of the ecto-kinase buffer containing 20 µM ATP and 50 µCi [γ-^32^P]ATP to CHO cells for 10 min at 37°C. Cells were lysed and immunoprecipitated for CD98. The immunoprecipitates were analyzed by SDS-PAGE, and radiolabeled bands were detected by autoradiography.

### Membrane extraction and Western blot analysis

CHO cells were scraped in cold PBS and collected by centrifugation at 2,000 *g* for 5 min. Cell pellets were suspended and homogenized in 5 mM HEPES pH 7.4 for 30 min at 4°C, and centrifuged at 13,000 *g* for 30 min at 4°C. The pellet was resuspended in PBS and saved as membrane extract. Membrane extracts were analyzed by SDS-PAGE and transferred to nitrocellulose membranes. Western blot was performed as previously described [16] using anti-V5 (Invitrogen), anti-CD98 (Santa Cruz), or anti-CD69 (Santa Cruz) antibody.

### Binding assay of Jurkat cells on Caco2-BBE monolayers

Confluent Caco2-BBE cells cultured in 96-well plates were incubated for 1 h at 37°C with 10 µg/ml of anti-CD98 antibody or the irrelevant IgG_1_ (Sigma). Cells were washed, and Jurkat cells in the ecto-kinase buffer containing 20 µM ATP were added to Caco2-BBE monolayers (10^6^ Jurkat cells/100 µl/well). Wells incubated with PBS were used as background. After a 30-min incubation at 37°C, the plate was washed with PBS and adherent cells were quantified with a colorimetric reaction by adding 100 µl hexosaminidase reagent (1% Triton X-100, 7.5 mM *p-*nitrophenyl *N*-acetyl-β-*D*-glucosaminide, in 100 mM natrium-citrate buffer pH 5.0) to each well for 40 min at 37°C. The reaction was stopped with 1 N NaOH. Absorbance was measured at 405 nm. Results were subtracted from background. Attached Jurkat cell numbers were determined from a standard curve generated using known Jurkat cell numbers.

### Binding assay of Jurkat cells on CHO cells

Jurkat cells were labeled with 5 µM 2′,7′-bis-(2-carboxyethyl)-5(-6)carboxyfluorescein, acetoxymethyl ester (BCECF-AM, Molecular Probes) in culture media for 30 min at 37°C. After washes, labeled Jurkat cells in the ecto-kinase buffer containing 20 µM ATP were added (10^7^ cells/400 µl/well) into CHO cells cultured in 12-well plates. Wells incubated with PBS were used as background. After a 30-min incubation at 37°C, wells were washed, and attached cells were lysed with 10 mM Tris, 150 mM NaCl, 3 mM EDTA and 1% Triton X-100. The fluorescence was read using a fluorescence spectrophotometer (Hitachi F-4500, Danbury, CT; excitation wavelength, 492 nm; emission wavelength, 520 nm). Results were subtracted from background. Numbers of attached Jurkat cells were determined based on calibration with lysates of BCECF-labeled Jurkat cells.

### Attachment assay of Caco2-BBE or Jurkat cells on recombinant proteins

Immulon 2HB 96-well high-binding plates (Thermo Scientific, Waltham, MA) were incubated for 3 h at 37°C with 10 µg/ml of recombinant CD98 or its mutated forms, or BSA used as a control. Wells incubated with PBS were used as background. The plate was washed with PBS and blocked with 3% BSA in PBS for 1 h at 37°C. Jurkat cells, pre-incubated with or without 10 µM K252b, or Caco2-BBE cells, suspended in serum-free media, were then added (2×10^5^ cells/100 µl/well) for 3 h at 37°C. Plates were rinsed with PBS, and adherent cells were quantified using the hexosaminidase-based colorimetric reaction as described above. Results were subtracted from background. Attached cell numbers were determined from standard curves generated using known numbers of Caco2-BBE or Jurkat cells.

### Cell adhesion measurement

Cell attachment and spreading were monitored using the electric cell-substrate impedance sensing (ECIS) 1600R device (Applied BioPhysics, Troy, NY). Caco2-BBE cells were seeded in ECIS electrodes pre-coated with 10 µg/ml of different proteins. Capacitance of cells was measured at 40 kHz, 1 V. The time necessary for cells to spread out on half of the electrode (*t*_1/2_) and the spreading rate of cells (*s*) were calculated for each electrode as previously described [18].

### Statistical analysis

Values are expressed as means±S.E.M. Statistical analysis was performed using unpaired Student's *t*-test. *P*<0.05 was considered statistically significant.

## Results

### CD98 is phosphorylated *in vitro* by CK2 and ePKs released from Jurkat cells

**Figure 1:**
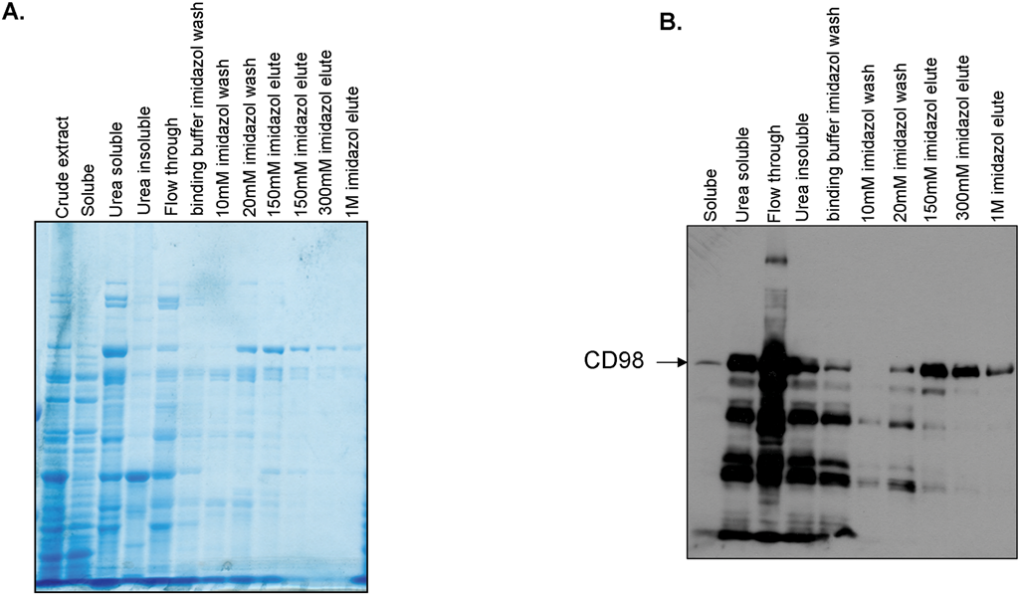
Purification of recombinant CD98. SDS-PAGE analysis and Comassie Blue gel staining (A) and Western blot analysis using anti-CD98 antibody (B) of recombinant CD98 purification.

**Figure 2:**
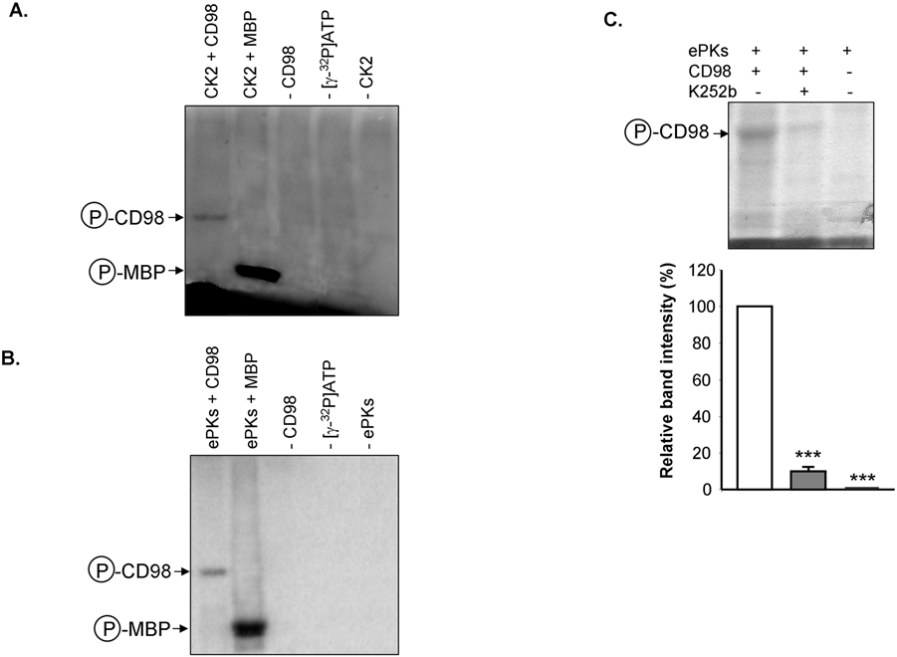
Recombinant CD98 is phosphorylated *in vitro* by casein kinase 2 and ecto-protein kinases from Jurkat cells. *In vitro* phosphorylation of 1 µg recombinant CD98 was performed using 2 µCi [γ-^32^P]ATP and 50 ng casein kinase 2 (CK2) (A) or 40 µl ecto-protein kinases (ePKs) purified from Jurkat cells (B). Control reactions were performed under the same condition except the absence of recombinant CD98, or [γ-^32^P]ATP, or CK2/ePKs, or the presence of 1 µg myelin basic protein (MBP). (C) Phosphorylation of recombinant CD98 by ePKs was performed in the presence or absence of the ePK inhibitor K252b. The samples were analyzed by SDS-PAGE, and phosphorylated proteins were detected by autoradiography. Bar graphs show the relative intensity of radiolabeled bands in the upper panel. Data are means±S.E.M of three determinations. ***P<0.001 *vs* white bar.

Analysis of the CD98 primary sequence using the NetPhos 2.0 server (http://www.cbs.dtu.dk/services/NetPhos/) indicated the presence of potential phosphorylation sites in its extracellular C-terminal tail. To determine whether CD98 is an *in vitro* substrate of ePKs, we first generated and purified recombinant CD98. The purity of recombinant CD98 was verified by SDS-PAGE (Fig. 1A) and Western blot (Fig. 1B). T cells have been found to possess ePKs [17], [19], [20], and it has been reported that incubation of intact cells with protein kinase substrates, such as casein, leads to the release of ePKs [15]. Here, Jurkat cells were incubated with 0.25 mg/ml dephosphorylated casein and ePKs released from the cells were purified. *In vitro* kinase assays were then performed using recombinant CD98, [γ-^32^P]ATP and Jurkat cell-derived ePKs or the commercial CK2. Autoradiographic analysis revealed phosphorylated CD98 bands from both phosphorylation reactions with CK2 (Fig. 2A) and with ePKs (Fig. 2B). As expected, there was no evidence of CD98 phosphorylation in control reactions performed in the absence of [γ-^32^P]ATP or CK2/ePKs (Fig. 2A and B). Furthermore, 10 µM of the ePK inhibitor K252b [21], [22], [23] significantly inhibited CD98 phosphorylation (Fig. 2C) but did not influence the total amounts of CD98 in the samples (data not shown). In addition, control reactions lacking recombinant CD98 did not show any phosphorylated band (Fig. 2A, B and C), indicating that the phosphorylation event was specific for CD98. Collectively, these results demonstrate that CD98 is phosphorylated *in vitro* by ePKs, including CK2.

**Figure 3:**
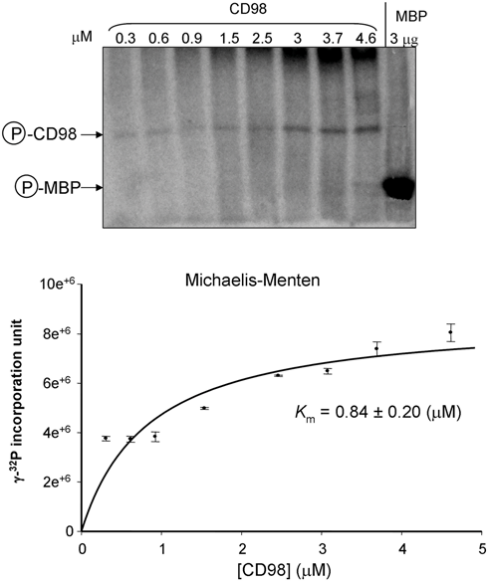
Kinetics of recombinant CD98 phosphorylation by casein kinase 2. Phosphorylation reactions were performed using different concentrations of recombinant CD98 and constant amounts of casein kinase 2 (CK2, 50 ng) and [γ-^32^P]ATP (2 µCi). Myelin basic protein (MBP), used as a positive control, was phosphorylated under the same condition. The samples were analysed by SDS-PAGE, and radiolabeled bands were detected and quantified using a Storm Typhoon 9200 PhosphorImager. The kinetic constant *K*_m_ was determined from a Michalis-Menten plot using an Enzyme Kinetic Program. Data are means±S.E.M of three determinations.

To determine the kinetic parameters of CK2-mediated CD98 phosphorylation, we performed *in vitro* kinase assays using increasing concentrations of recombinant CD98 and constant amounts of CK2 and [γ-^32^P]ATP. The samples were analyzed by SDS-PAGE, and radiolabeled bands were detected by autoradiography (Fig. 3, upper panel). The kinetic constant *K*_m_ was determined from a Michalis-Menten plot using the Enzyme Kinetic Program. As summarized in Figure 3, kinetic analysis of the *in vitro* phosphorylation of recombinant CD98 by CK2 revealed a *K*_m_ = 0.84±0.20 (µM) for CD98. This *K*_m_ is within the range of protein phosphorylation by CK2 [11].

### Serines-305/307/309 and serines-426/430 of CD98 are the dominant phosphorylation sites for CK2

**Figure 4:**
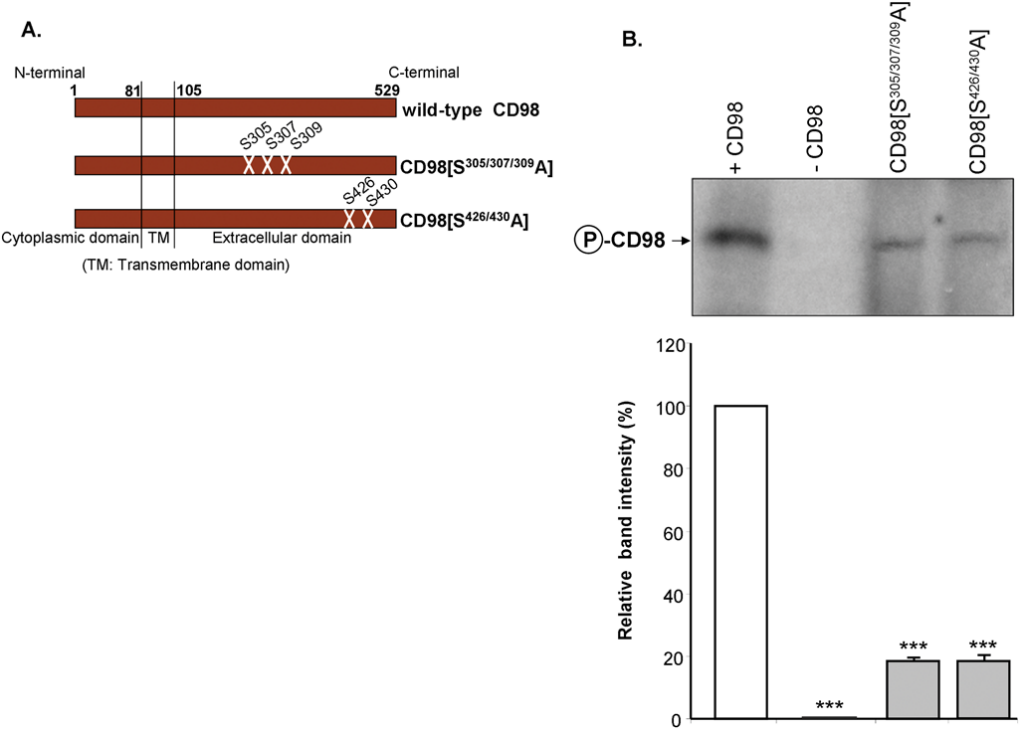
Serines-305/307/309 and serines-426/430 of CD98 are the dominant phosphorylation sites for casein kinase 2. (A) Schematic representation of the variant CD98 constructs. (B) *In vitro* phosphorylation of 1 µg of recombinant wild-type CD98 or its mutated forms CD98[S^305/307/309^A] and CD98[S^426/430^A] was performed using 2 µCi [γ-^32^P]ATP and 50 ng casein kinase 2 (CK2) at 30°C for 10 min. A reaction performed in parallel in the absence of recombinant CD98 (-CD98) was used as a negative control. The samples were analysed by SDS-PAGE. Radiolabeled bands were detected by autoradiography. Bar graphs show the relative intensity of radiolabeled bands in the upper panel. Data are means±S.E.M of three determinations. ***P<0.001 *vs* +CD98 (white bar).

To further explore the ecto-domain of CD98, we generated CD98 constructs mutated at the potential phosphorylation sites serines-305/307/309 or serines-426/430 (Fig. 4A); these sites were predicted with high score (S^305^: 0.99; S^307^: 0.97; S^309^: 0.98; S^426^: 0.99; S^430^: 0.99) by the NetPhos 2.0 Server. Recombinant wild-type CD98 or its mutated forms, CD98[S^305/307/309^A] and CD98[S^426/430^A], were used as substrates for *in vitro* kinase assays using CK2 and [γ-^32^P]ATP. Figure 4B shows that substitutions of serines-305/307/309 or serines -426/430 by alanine residues attenuated CD98 phosphorylation by ∼78 and 79%, respectively. The control reaction performed in the absence of recombinant CD98 did not induce any phosphorylation (Fig. 4B, lane 2). Together, these results suggest that serines-305/307/309 and serines-426/430 are the dominant CD98 phosphorylation sites for CK2.

### CD98 expressed in Caco2-BBE cell membranes is ecto-phosphorylated by Jurkat cell-derived ePKs

**Figure 5:**
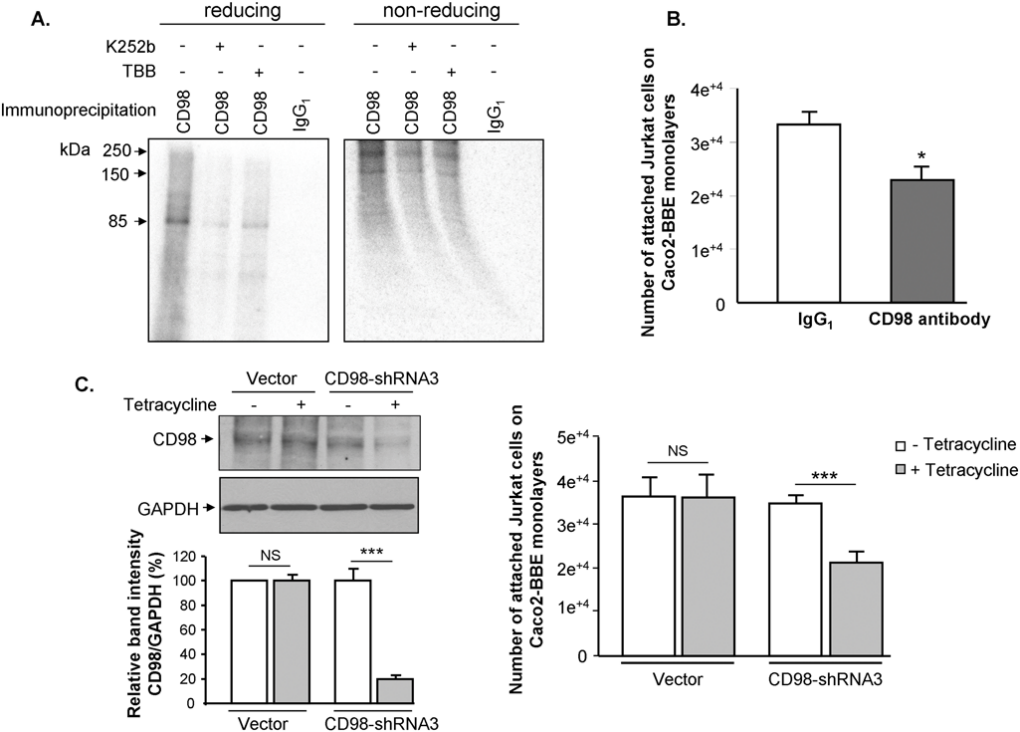
CD98 in Caco2-BBE cells is phosphorylated by ecto-protein kinases from Jurkat cells and its role in epithelial cell-lymphocyte interactions. (A) The basolateral surface of Caco2-BBE monolayers, grown on filters, was exposed to a reaction mixture containing ecto-protein kinases purified from Jurkat cells and 50 µCi [γ-^32^P]ATP for 10 min at 37°C. Inhibition kinase assays were performed in the presence of 10 µM of the broad-spectrum ecto-protein kinase inhibitor K252b or 10 µM of the CK2-specific inhibitor 4,5,6,7-Tetrabromobenzotriazole (TBB). Cell lysates were immunoprecipitated with anti-CD98 antibody or the irrelevant IgG_1_. The immunoprecipitates were analyzed by SDS-PAGE under reducing or non-reducing conditions. Radiolabeled bands were detected by autoradiography. (B) Binding assays of Jurkat cells on Caco2-BBE monolayers were performed under the ecto-kisase assay condition as described in Materials and Methods. Caco2-BBE cells cultured in 96-well plates were pre-incubated for 1 h at 37°C with 10 µg/ml of CD98 antibody or the control IgG_1_, and Jurkat cells were added (10^6^ Jurkat cells/100 µl/well) for 40 min at 37°C. Adherent cells were quantified by hexosaminidase-based colorimetric reaction. Data were subtracted from background, and the numbers of attached Jurkat cells were determined from a standard curve generated using known numbers of Jurkat cells. (C) *Tet*R Caco2-BBE cells transfected with the CD98-shRNA3 construct or the empty vector were treated or not with 2.5 µg/ml tetracycline for 2 days and analysed for CD98 expression by Western blot (left panel), or were used for cell binding assay as described in (B) (right panel). Bar graphs show the relative intensity of bands in the upper panel. Data are means±S.E.M of three determinations. *P<0.05; ***P<0.001; NS, not statically significant.

Having found that recombinant CD98 is phosphorylated by ePKs *in vitro*, we then sought to determine whether CD98 expressed in the basolateral membranes of intestinal epithelial Caco2-BBE cells is phosphorylated by ePKs. To accomplish this, the basolateral surface of Caco2-BBE monolayers, grown on filters, was exposed to a reaction mixture containing Jurkat cell-derived ePKs and [γ-^32^P]ATP for 10 min at 37°C. For inhibition study, the reactions were performed in the presence of the broad-spectrum ePK inhibitor K252b or the CK2-specific inhibitor TBB. The cells were lysed and immunoprecipitated for CD98 or the irrelevant IgG_1_. Autoradiographic analysis of CD98 immunoprecipitate resolved by SDS-PAGE under reducing condition revealed an 85-kDa phosphorylated band that represents glycosylated CD98 (Fig. 5A, left panel). Under non-reducing conditions, CD98 immunoprecipitate migrated as two radiolabeled bands of ∼130 and ∼250 kDa (Fig. 5A, right panel). The 130-kDa band represents the heterodimer of CD98 and the amino-acid transporter LAT-2 as we have previously shown [5], whereas the 250-kDa band could be an association of CD98/LAT-2 heterodimer with other molecules. In the control IgG_1_ immunoprecipitate, however, no radiolabeled band was detected under either reducing or non-reducing condition (Fig. 5A). In addition, 10 µM of the cell-impermeable broad-spectrum ePK inhibitor K252b markedly inhibited CD98 phosphorylation under both reducing and non-reducing conditions, demonstrating that CD98 was ecto-phosphorylated by ePKs (Fig. 5A). To test if CK2 is involved in the ecto-phosphorylation of CD98, phosphorylation reaction was performed in the presence of 10 µM of TBB, a CK2-specific inhibitor [24], [25]. As shown in Figure 5A, TBB effectively inhibited CD98 phosphorylation, indicating the involvement of ecto-CK2 in this process. Collectively, these results suggest that CD98 basolaterally expressed in Caco2-BBE monolayers is ecto-phosphorylated by ePKs, including ecto-CK2, in its physiological setting, where it may associate with other molecules to form multi-component complexes.

### CD98 plays a role in epithelial cell-T lymphocyte interactions

Having shown that CD98 in Caco2-BBE cells is phosphorylated by ePKs from Jurkat cells, we then asked if its phosphorylation could be involved in interactions between IECs and T lymphocytes. To address this possibility, we examined the adhesion of Jurkat cells on Caco2-BBE monolayers under the ecto-kinase condition as described in Materials and Methods. Caco2-BBE monolayers, cultured in 96-well plates, were pre-incubated with CD98 antibody or the irrelevant IgG_1_, and Jurkat cells were added. We found that incubation of Caco2-BBE cells with CD98 antibody significantly reduced Jurkat cell binding by ∼31% (Fig. 5B), suggesting a role for CD98 and/or its phosphorylation in IEC-T lymphocyte interactions.

To further study the role of CD98 in cell-cell interactions, we modulated CD98 protein expression in Caco2-BBE cell lines using a *Tet*-off system and studied the interactions between these cells and T lymphocytes. Western blot analyses of *Tet*R Caco2-BBE cells transfected with three CD98-shRNA constructs after 2, 4 or 6 days of treatment with 2.5 or 5 µg/ml tetracycline revealed that only the CD98-shRNA3 construct (see Materials and Methods) was effective in knocking down CD98, with highest repression effect after 2 days of 2.5 µg/ml tetracycline treatment (Fig. 5C, left panel). Interestingly, the CD98 silencing in Caco2-BBE cells consequently reduced Jurkat cell binding by 39% (Fig. 5C, right panel). In contrast, tetracycline treatment (2.5 µg/ml, 2 days) of *Tet*R Caco2-BBE cells transfected with the empty vector did not have any significant effect on CD98 expression and Jurkat cell binding. Collectively, these results point out the importance of epithelial CD98 and/or its phosphorylation in IEC-T lymphocyte interactions.

### Ecto-phosphorylation of CD98 expressed in CHO cells at serines-305/307/309 and serines-426/430 is crucial for Jurkat cell-CHO cell interactions

**Figure 6:**
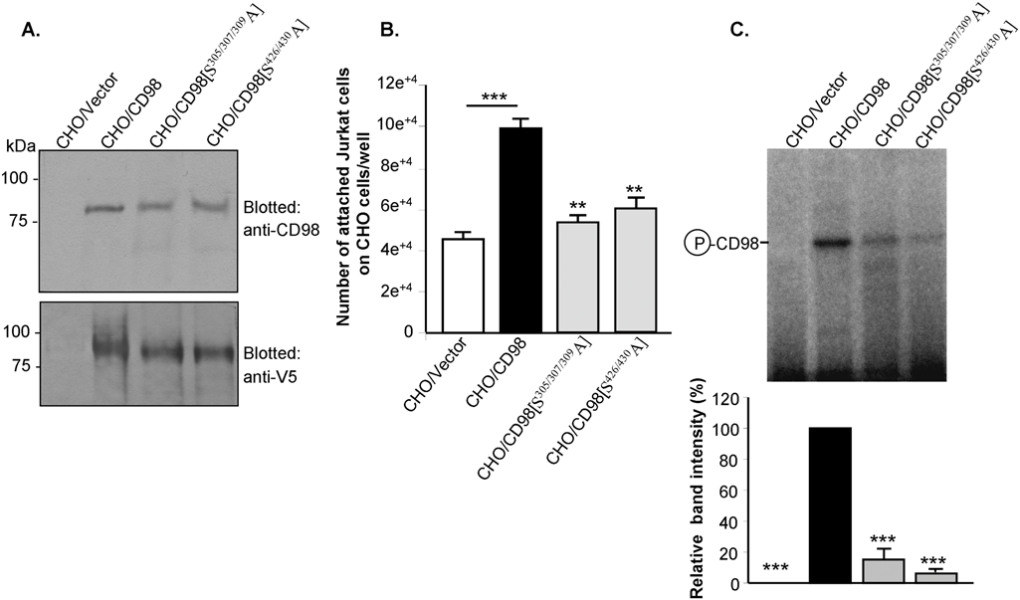
Ecto-phosphorylation of CD98 in CHO cell membranes enhances Jurkat cell-CHO cell interactions. CHO cells were transfected with the pcDNA3.1 TOPO/V5 vector (CHO/Vector) or V5-fused constructs of wild-type CD98 (CHO/CD98) or CD98 mutated at serines-305/307/309 (CHO/CD98[S^305/307/309^A]) or sesrines-426/430 (CHO/CD98[S^426/430^A]). (A) Western blot analysis of membrane extracts from transfected CHO cells using anti-CD98 or anti-V5 antibody (upper panel). (B) Cell binding assays of fluorescent-labeled Jurkat cells on transfected CHO cells were performed under the ecto-kinase assay condition as described in Materials and Methods. Data are means±S.E.M of three independent determinations. **P<0.005 vs CHO/CD98; ***P<0.001. (C) Ecto-phosphorylation of CD98 expressed in CHO cells by intact Jurkat cells. Ecto-kinase assays were performed by applying Jurkat cells in the ecto-kinase buffer containing 10 µCi [γ-^32^P]ATP to CHO cells for 10 min at 37°C. After washes, CHO cells were lysed, immunoprecipitated for CD98 and analysed by SDS-PAGE. Radiolabeled bands were detected by autoradiography. Bar graphs show the relative intensity of radiolabeled bands in the upper panel. Data are means±S.E.M of three determinations. ***P<0.001 vs CHO/CD98.

To confirm the functional involvement of CD98 phosphorylation in cell-cell interactions, we expressed variant CD98 constructs (Fig. 4A) in CHO cells, which normally do not express CD98, and examined the interactions between these cells and T lymphocytes. CHO cells were transfected with the pcDNA3.1 TOPO/V5 vector (CHO/Vector), or the cDNA encoding wild-type CD98 (CHO/CD98) or phosphorylation-deficient CD98 mutants (CHO/CD98[S^305/307/309^A] and CHO/CD98[S^426/430^A]) fused with V5 protein. Western blot analyses using anti-CD98 or anti-V5 antibody showed that exogenous wild-type and mutated CD98 proteins were successfully expressed in CHO cell membranes (Fig. 6A). The role of CD98 phosphorylation in cell-cell interactions was assessed by measuring adhesion of Jurkat cells, in the ecto-kinase buffer containing non-radioactive ATP, on confluent transfected CHO cells. Figure 6B shows that over-expression of CD98 in CHO cells induced a marked increase (∼2.2-fold) in Jurkat cell binding to CHO cells. Interestingly, the numbers of Jurkat cells attached on CHO/CD98[S^305/307/309^A] and CHO/CD98[S^426/430^A] were significantly decreased by ∼1.9- and 1.7-fold, respectively, compared to that on CHO/CD98 (Fig. 6B). To verify if CD98 expressed in CHO cells is ecto-phosphorylated by Jurkat cells under this adhesion assay condition, we performed an ecto-kinase assay by applying intact Jurkat cells in the ecto-kinase buffer containing [γ-^32^P]ATP to CHO cells for 10 min at 37°C. Figure 6C shows a radiolabeled band in CD98 immunoprecipitate derived from CHO/CD98, demonstrating that CD98 in these cells was ecto-phosphorylated. The incorporation of radioactive phosphate groups into CD98[S^305/307/309^A] and CD98[S^426/430^A] expressed in CHO cell membranes was remarkably suppressed by ∼84 and 95%, respectively, compared to that to wild-type CD98 (Fig. 6C). Collectively, these findings confirm the ecto-phosphorylation of CD98 localized on the cell surface and pointed out that such phosphorylation plays an important role in heterotypic cell-cell interactions.

### The C-terminal extracellular domain of CD98 is important for cell-cell interactions

**Figure 7:**
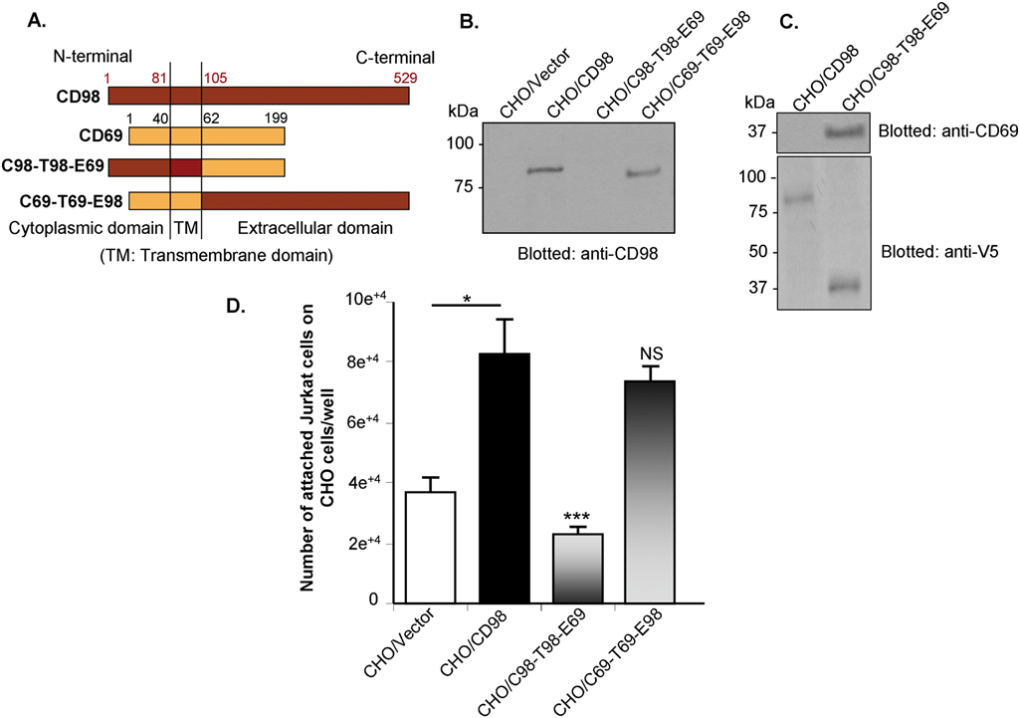
The extracellular C-terminal tail of CD98 expressed in CHO cell membranes is important for Jurkat cell-CHO cell interactions. (A) Schematic representation of variant CD98 constructs used in this study: chimeras of CD98 with CD69 (C98-T98-E69 contains the cytoplasmic and transmembrane domains of CD98 and the extracellular domain of CD69; C69-T69-E98 contains the cytoplasmic and transmembrane domains of CD69 and the extracellular domain of CD98). (B) and (C) Western blot analysis using anti-CD98 (B), anti-CD69 (C) or anti-V5 (C) antibody of membrane extracts from CHO cells stably transfected with the variant constructs. (D) Cell binding assays of fluorescent-labeled Jurkat cells on transfected CHO cells under the ecto-kinase assay condition as described in Materials and Methods. Data are means±S.E.M of three determinations. *P<0.05; (***P<0.001; NS, not statically significant) *vs* CHO/CD98.

To further study the molecular mechanisms underlying CD98-mediated cell-cell interactions, we generated chimeras of CD98 with CD69: C98-T98-E69 contains cytoplasmic and transmembrane domains of CD98 and the extracellular domain of CD69; C69-T69-E98 contains cytoplasmic and transmembrane domains of CD69 and the extracellular domain of CD98 (Fig. 7A). Wild-type CD98 and the chimeras, subcloned into pcDNA3.1 TOPO/V5 vector, were stably transfected into CHO cells. Western blot analyses of membrane extracts from transfected CHO cells using anti-V5, anti-CD98 or anti-CD69 antibody were performed to examine the expression of exogenous proteins. Blotting with anti-CD98 antibody, raised against an epitope in the C-terminus of CD98, revealed the expression of wild-type CD98 and C69-T69-E98 (Fig. 7B). However, this antibody could not recognize C98-T98-E69, which lacks the C-terminus of CD98 (Fig. 7B, lane 4). The expression of this protein was confirmed by immunoblotting with anti-CD69 antibody (Fig. 7C, upper panel), which recognizes an epitope in the C-terminus of CD69, or anti-V5 antibody (Fig. 7C, lower panel), which recognizes the fusion variants. Adhesion assays of Jurkat cells on the transfected CHO cells were then performed under the ecto-kinase assay condition. In agreement with the data shown in Figure 6B, over-expression of CD98 in CHO cells increased Jurkat cell binding by ∼2.2 fold (Fig. 7D). Deletion of the extracellular domain of CD98 (on CHO/C98-T98-E69) abrogated this CD98-mediated increase in Jurkat cell binding, reducing the numbers of attached Jurkat cells by ∼72% relative to that on CHO/CD98 (Fig. 7D). In contrast, Jurkat cell attachment on CHO/C69-T69-E98, which lacks N-terminus of CD98, was not significantly different from that on CHO/CD98 (Fig. 7D). These data indicate that the C-terminal extracellular domain of CD98 is important for cell-cell interactions.

### Recombinant CD98 interacts with Caco2-BBE cells, enhancing epithelial cell adhesion

**Figure 8:**
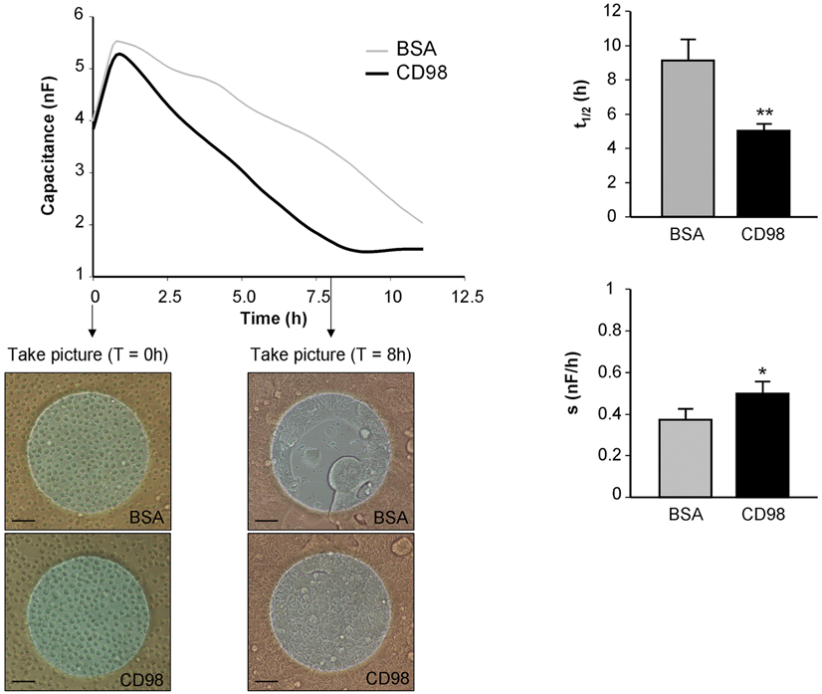
Recombinant CD98 interacts with Caco2-BBE cells and enhances epithelial cell attachment and spreading. Caco2-BBE cell attachment was monitored in real-time using the electric cell-substrate impedance-sensing (ECIS) device. Caco2-BBE cells were seeded on electrodes coated with 10 µg/ml of recombinant CD98 or BSA (2×10^5^ cells/electrode). Capacitance was measured at 40 kHz, 1 V. Microscopic images were taken at *t*_0_ and 8-h post-seeding. Each image is a representative of quadruplicate electrodes. Bars, 50 µm. Half time (*t*_1/2_) and spreading rate (*s*) of the cells were determined for each electrode. Data are means±S.E.M of three determinations. *P<0.05; **P<0.005.

Having established the importance of CD98 in cell-cell interactions, we next turned our interest into the role of CD98 in cell adhesion. Attachment and spreading of Caco2-BBE cells were monitored quantitatively and in real time using the electric cell-substrate impedance sensing (ECIS) technique. Capacitance of cells at 40 kHz was measured on electrodes coated with 10 µg/ml of recombinant CD98 or the control protein BSA. The time necessary for cells to spread out on half of the available electrode (*t*_1/2_) and the spreading rate of cells (*s*) were accordingly determined. Figure 8 shows that Caco2-BBE cells attached and spread significantly faster on CD98 (black line: *t*_1/2_ = 5±0.46 h; *s* = 0.5±0.06 nF/h) than on BSA (grey line: *t*_1/2_ = 9.12±1.65 h; *s* = 0.38±0.02 nF/h). Furthermore, microscopic images taken 8-h post-seeding show that Caco2-BBE monolayers on CD98-coated electrodes were completely confluent, whereas those on BSA-coated electrodes were not (Fig. 8), supporting the capacitance measurement and spreading rate data. Together, these results demonstrate that recombinant CD98 interacts with Caco2-BBE cell membranes and such interaction enhances attachment and spreading of IECs.

### Phosphorylation of recombinant CD98 increases its interaction with Caco2-BBE cells and promotes epithelial cell adhesion

**Figure 9:**
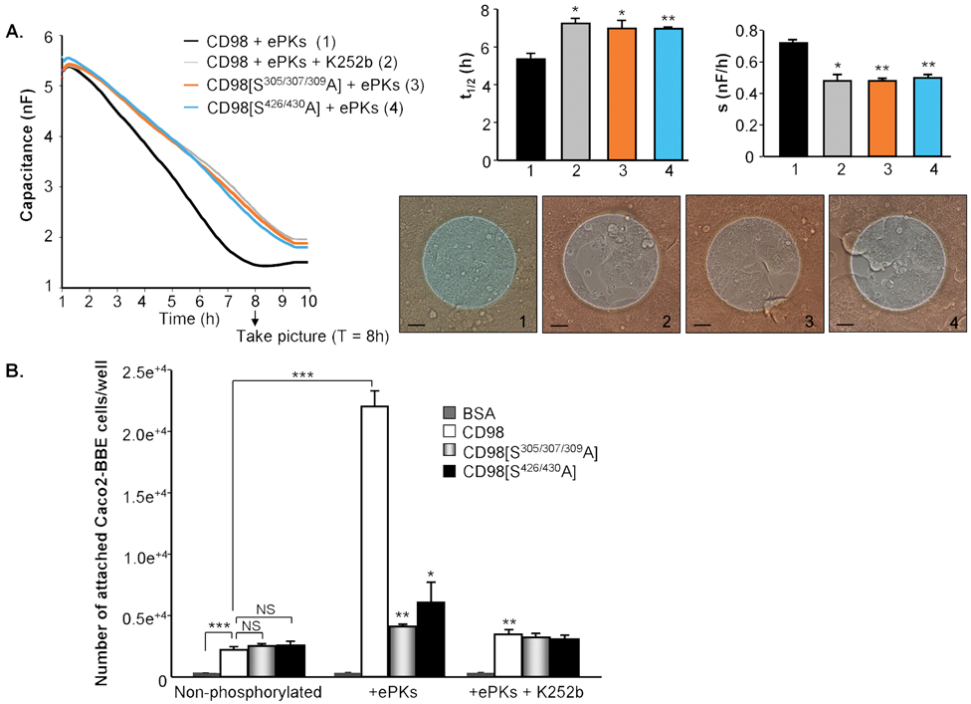
Phosphorylation of recombinant CD98 increases its interaction with Caco2-BBE cells and promotes epithelial cell adhesion. (A) Recombinant wild-type CD98 or its mutated forms CD98[S^305/307/309^A] and CD98[S^426/430^A] (2 µg each) were applied to *in vitro* kinase assays using 50 µM ATP and ecto-protein kinases (ePKs) purified from Jurkat cells in the presence or absence of 10 µM of the ePK inhibitor K252b. Caco2-BBE cells were seeded on the electric cell-substrate impedance-sensing electrodes coated with these samples (2×10^5^ cells/electrode). Capacitance was measured at 40 kHz, 1 V. Each microscopic image taken 8-h post-seeding is a representative of quadruplicate electrodes. Bars, 50 µm. Half time (*t*_1/2_) and spreading rate (*s*) of the cells were determined for each electrode. Data are means±S.E.M of three determinations. (*P<0.05; **P<0.005) *vs* CD98+ePKs (black bar). (B) Cell attachment assays were performed in 96-well high-binding plates. Wells were coated with 10 µg/ml of BSA (control), recombinant wild-type CD98 or its mutated form CD98[S^305/307/309^A] and CD98[S^426/430^A]. Some wells were coated with the proteins pre-applied to *in vitro* kinase assays with ePKs from Jurkat cells in the presence or absence of K252b. After incubation for 3 h at 37°C, the plate was washed and blocked for 1 h at 37°C. Caco2-BBE cells were added (2×10^5^ cells/well) for 3 h at 37°C. After washes, attached cells were quantified by a hexosaminidase-based calorimetric reaction. Data were subtracted from background, and the numbers of attached cells were determined from a standard curve generated using known numbers of Caco2-BBE cells. Data are means±S.E.M of three determinations. (*P<0.05; **P<0.005) *vs* CD98+ePKs; ***P<0.001; NS, not statically significant.

The finding that CD98 interacts with Caco2-BBE cells led us to hypothesize that its phosphorylation could affect the attachment and spreading of IECs. To test this possibility, we compared Caco2-BBE cell adhesion on phosphorylated CD98 with that on deficiently phosphorylated CD98. Recombinant wild-type CD98 and the phosphorylation-deficient mutants CD98[S^305/307/309^A] and CD98[S^426/430^A] were applied to *in vitro* kinase assays with ePKs in the presence or absence of K252b, and cell adhesion on these substrates was monitored using the ECIS device. Figure 9A shows that K252b, which effectively inhibited CD98 phosphorylation *in vitro* (Fig. 2C), reduced cell adhesion on phosphorylated CD98 (on CD98+ePKs+K252b[grey line]: *t*_1/2_ = 7.25±0.25 h, *s* = 0.48±0.04 nF/h *vs* on CD98+ePKs[black line]: *t*_1/2_ = 5.35±0.3 h, s = 0.72±0.02 nF/h). Furthermore, cell adhesion on phosphorylated CD98 was significantly faster than that on either CD98[S^305/307/309^A]+ePKs (orange line: *t*_1/2_ = 7±0.39 h; *s* = 0.48±0.02 nF/h) or on CD98[S^426/430^A]+ePKs (blue line: *t*_1/2_ = 6.95±0.1 h; *s* = 0.5±0.02 nF/h) (Fig. 9A). Microscopic images show that Caco2-BBE monolayers reached confluency at 8-h post-seeding on (CD98+ePKs)-coated electrodes, but were not confluent on electrodes coated with CD98+ePKs+K252b, CD98[S^305/307/309^A]+ePKs or CD98[S^426/430^A]+ePKs (Fig. 9A). These data strongly suggest that the phosphorylation of CD98 at both phosphorylation sites, serines-305/307/309 and serines-426/430, contribute to the adhesive function of CD98.

Similar results were obtained from cell attachment assay performed in 96-well high-binding plates. Plates were coated with recombinant CD98, CD98[S^305/307/309^A] or CD98[S^426/430^A] (non-phosphorylated; Fig. 9B), or with the proteins pre-applied to *in vitro* kinase assays in the absence (+ePKs) or presence of K252b (+ePKs+K252b) (Fig. 9B). Figure 9B shows a higher number of Caco2-BBE cells attached on recombinant CD98 (2,200±260 cells/well) than on the control BSA (63±5 cells/well), suggesting a role of CD98 in cell attachment. Remarkably, ePK-mediated phosphorylation of CD98 enhanced cell attachment by ∼10-fold (22,005±3,500 cells/well on CD98+ePKs *vs* 2,200±260 cells/well on non-phosphorylated CD98; Fig. 9B). As expected, cell attachment on CD98[S^305/307/309^A] or CD98[S^426/430^A] was not significantly increased when these phosphorylation-deficient mutated proteins were pre-incubated with ePKs (Fig. 9B). The numbers of cells binding on CD98[S^305/307/309^A]+ePKs and CD98[S^426/430^A]+ePKs were decreased by ∼81 and 72%, respectively, compared to that on CD98+ePKs, although cell attachment on non-phosphorylated CD98, CD98[S^305/307/309^A] and CD98[S^426/430^A] was similar (Fig. 9B). Furthermore, suppression of CD98 phosphorylation by K252b decreased cell attachment by ∼84% (3,500±380 cells/well on CD98+ePKs+K252b *vs* 22,005±3,500 cells/well on CD98+ePKs; Fig. 9B). These observations strongly support the finding that CD98 phosphorylation plays an important role in Caco2-BBE cell adhesion.

Collectively, our results, obtained from Caco2-BBE cell attachment measurements using the ECIS device and high-binding plates, indicate that the phosphorylation of CD98, dominantly at serines-305/307/309 and serines-426/430, enhances its interaction with IECs, promoting epithelial cell attachment and spreading.

### Phosphorylation of recombinant CD98 increases its interaction with Jurkat cells and promotes cell attachment

**Figure 10:**
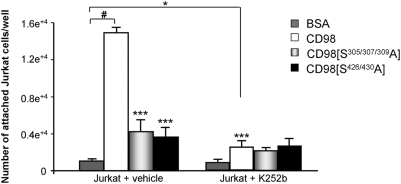
Phosphorylation of recombinant CD98 increases its interaction with Jurkat cells and enhances cell attachment. Attachment of Jurkat cells was assayed in 96-well high-binding plates coated with 10 µg/ml of BSA (control), recombinant wild-type CD98 or its mutated forms CD98[S^305/307/309^A] and CD98[S^426/430^A] for 3 h at 37°C. The plate was washed and blocked for 1 h at 37°C. 2×10^5^ Jurkat cells, pre-incubated without (Jurkat+vehicle) or with 10 µM of the ecto-protein kinase inhibitor K252b for 30 min at 37°C (Jurkat+K252b), were added to each well for 3 h at 37°C. After washes, attached cells were quantified using a hexosaminidase-based calorimetric method. Results were subtracted from background. The numbers of attached cells were determined from a standard curve generated using known numbers of Jurkat cells. Data are means±S.E.M of three determinations. ***P<0.001 *vs* (Jurkat+vehicle) on CD98; ^#^ *P*<0.001;^ *^ *P* <0.05.

To investigate whether CD98 interacts with Jurkat cells and if yes how phosphorylation of CD98 affects this interaction, we performed cell attachment assays in 96-well plates coated with recombinant wild-type CD98 or its mutated forms. Figure 10 shows that Jurkat cells attached better on wild-type CD98 (14,931±1,670 cells/well) than on BSA (1,020±800 cells/well) or on the phosphorylation-deficient mutants CD98[S^305/307/309^A] (4,189±1,382 cells/well) and CD98[S^426/430^A] (3,640±1,010 cells/well). These data suggest that ePKs could be released from Jurkat cells during the incubation of cells with CD98, leading to CD98 phosphorylation and, consequently, an increase in Jurkat cell attachment. Furthermore, K252b markedly decreased the binding of Jurkat cells to CD98 by 83% (2,505±647 Jurkat cells+K252b/well *vs* 14,931±577 Jurkat cells/well), but did not significantly affect cell binding to CD98[S^305/307/309^A] or CD98[S^426/430^A] (Fig. 10). Together, these data demonstrate that CD98 interacts with Jurkat cells and this interaction is enhanced by the phosphorylation of CD98.

## Discussion

The present study shows that CD98 is phosphorylated and demonstrates that the ecto-phosphorylation of CD98 is functionally important in cell-cell interactions. Our study has three major findings. First, recombinant CD98 is phosphorylated *in vitro* by CK2 and by ePKs from T lymphocytes. Second, CD98 basolaterally expressed in IECs can be ecto-phosphorylated at its extracellular C-terminus, dominantly at serines-305/307/309 and serines-426/430. Third, CD98 is involved in IEC adhesion, and phosphorylation of CD98 enhances cell adhesion and cell-cell interactions. Our findings suggest that the selective induction of CD98 phosphorylation by ePKs is a pivotal event in IEC-T lymphocyte interactions.

The cell surface is directly involved in cell-cell interactions through receptors for extracellular signals. The phosphorylation and dephosphorylation of membrane proteins play an important role in the regulation of many cellular functions [7]. For example, T cell surface receptors, including cell adhesion proteins and recognition molecules, are regulated through phosphorylation of their extracellular domains [17], [19], [26], [27]. CD98, originally discovered as a surface antigen in lymphocytes [2], has been found to be important in many biological processes, such as cell adhesion, amino-acid transport and signal transduction [1]. The presence of a C-terminal extracellular domain with potential phosphorylation sites led us to propose that CD98 could be extracellularly phosphorylated and such phosphorylation could regulate its multiple functions.

Using kinase assays with extensively controlled conditions, we showed that ePKs from Jurkat cells were as effective as the commercial CK2 in phosphorylating recombinant CD98 *in vitro*. CK2 is a ubiquitous and highly conserved serine/threonine kinase [28] that has been reported to phosphorylate hundreds of putative physiological substrates. The minimal consensus sequence for CK2-mediated phosphorylation has been identified as S/T-X-X-Acidic [29], [30], [31] where the +3 acidic residue can be Asp, Glu, pSer [32], [33] or pTyr [34], but apparently not pThr [32], [34]. These observations, together with information given by NetPhos 2.0 Server, enabled us to identify serines-305/307/309 and serines-426/430, located in the extracelullar domain of CD98, as potential CK2 phosphorylation sites. We showed here that alanine substitutions of serines-305/307/309 or serines-426/430 markedly reduced the incorporation of γ-^32^P into CD98 by CK2 *in vitro* and attenuated the phosphorylation of CD98 in CHO cell membranes by ePKs, indicating that these residues are the dominant phosphorylation sites. The exact phosphorylation sites in CD98 molecule remain within the scope of further investigations.

Importantly, we demonstrated that CD98 basolaterally expressed in the intestinal epithelial Caco2-BBE cells is phosphorylated by ePKs from Jurkat cells, and that ecto-CK2 is involved in this process. Interestingly, CD98 is ecto-phosphorylated in its physiological setting, where it associates with other molecules to form multi-component complexes. We have previously reported that CD98 forms heterodimers with amino-acid transporters, and these heterodimers interact with β_1_-integrin [5] and the intercellular adhesion molecule ICAM-1 [35] in the basolateral membranes of IECs. The ecto-phosphorylation of CD98 investigated here is of utmost importance since it represents a novel mechanism by which CD98 may be involved in phosphorylation-dependent transduction of extracellular signals into the cells.

The major finding of our study is that ecto-phosphorylation of CD98 plays an important role in cell-cell interactions. Results from cell attachment assays suggested that ecto-phosphorylation of CD98 in Caco2-BBE cells was involved in Caco2-BBE cell-Jurkat cell interactions. Furthermore, ecto-phosphorylation of CD98 expressed in CHO cells increased the binding of Jurkat cells to CHO cells, and mutation at serines-305/307/309 or serines-426/430 attenuated CD98 phosphorylation by Jurkat cells and consequently reduced cell-cell interactions. Importantly, we demonstrated that the C-terminal extracellular domain of CD98, which possesses phosphorylation sites, was crucial for the heterotypic cell-cell interactions. Together, these findings suggest that the phosphorylation of CD98 on its extracellular domain by ePKs from T lymphocytes is important for epithelial cell-T lymphocyte interactions. In addition, applying the ECIS technology to monitor IEC adhesion on different substrates in real time, we demonstrated that attachment and spreading of Caco2-BBE cells on recombinant CD98 were enhanced when CD98 was phosphorylated. In agreement with the ECIS data, cell adhesion assays performed in 96-well high-binding plates showed that the attachment of Caco2-BBE and Jurkat cells was increased on phosphorylated recombinant CD98. Mutation at both phosphorylation sites, serines-305/307/309 and serines-426/430, abolished the effect of ePK-mediated CD98 phosphorylation on cell adhesion, further demonstrating the contribution of phosphorylation to the adhesive function of this molecule. These results strongly suggest that the phosphorylation of CD98 could modulate its specific ability to interact with membranes of other cells, which might account for homotypic and/or heterotypic cell-cell interactions.

Previous sdudies have demonstrated that several proteins that are involved in cell adhesion can be extracellularly phosphorylated and that their ecto-phosphorylation can regulate cell adhesion and cell migration. It has been reported that vitronectin, an extracellular maxtrix glycoprotein, is phosphorylated by CK2 on the surface of blood cells, and that cell adhesion on CK2-phosphorylated vitronectin is enhanced via the alpha(v)beta3 integrin [36]. Ecto-CK2-mediated vitronectin phosphorylation was also shown to be important to vascular smooth muscle cell adhesion [37]. Similarly, laminin-1 can be phosphorylated by CK2-type ePKs of monocyte, and its phosphorylation increases cell adhesion, cell proliferation as well as cell migration [21]. Recently, the cell surface receptor collagen XVII was identified as a substrate of ecto-CK2 [38]. Ecto-phosphorylation of collagen XVII was also demonstrated to inhibit its ecto-domain shedding, which is involved in the regulation of adhesion, motility and differentiation of epithelial cells [38]. Together with these investigations, our finding that ecto-phosphorylation of CD98 promotes cell adhesion suggest that the extracellular phosphorylation of cell-surface proteins and extracellular matrix proteins seems to be the general mechanism underlying the regulation of cell adhesion.

The involvement of CD98 ecto-phosphorylation in cell-cell interactions found here is of great importance from a pathophysiological perspective because the interactions between IECs and lymphocytes play a key role in immune responses within the intestinal tract [39]. It is interesting to speculate that under pathological conditions, where extracellular ATP required for ecto-phosphorylation can reach high concentration [7], CD98 phosphorylation is especially effective, which affects cell-cell interactions and consequently regulates T cell-mediated immune responses. Further studies are required to investigate which physiological and/or pathological state(s) can actuate this mechanism. Previous studies have suggested a role for CD98 in the etiology of inflammatory disorders. Pro-inflammatory cytokines have been shown to upregulate CD98 expression in IECs [14], [40]. Increased levels of lymphocyte-activation antigens, including CD98, have been found at the cell surface of intestinal B cells, CD4^+^ T cells and CD8^+^ T cells isolated from patients with inflammatory bowel disease [41]. We have recently showed that CD98 expression is highly upregulated in colonic tissues from mice with active colitis, and that activation of epithelial CD98 aggravates intestinal inflammation [42]. These studies and our present findings suggest that CD98 could be a potential pharmacological target to manipulate lymphocyte activation and IEC-lymphocyte interactions during intestinal inflammation.

The molecular mechanism by which the ecto-phosphorylation of CD98 modulates cell-cell interactions remains to be elucidated. It is tempting to speculate that CD98 ecto-phosphorylation could have substantial effects on the binding affinity of CD98 for proteins expressed on the surface of other cells. However, we cannot exclude the possibility that the phosphorylation of CD98 may functionally affect other proteins that interact with CD98 and are involved in cell-cell interactions.

In conclusion, our study demonstrates that CD98 is ecto-phosphorylated by ePKs, including ecto-CK2, dominantly at serine-305/307/309 and serine-426/430, and that the ecto-phosphorylation of CD98 plays an important role in cell-cell interactions. Our findings reveal a novel mechanism underlying the regulation of the multiple functions of CD98 and suggest that CD98 could be a promising target for therapeutic strategies to prevent aberrant cell-cell interactions, which have been implicated in the pathogenesis of various intestinal diseases.

## References

[1] Yan Y, Vasudevan S, Nguyen H, Merlin D (2008). Intestinal epithelial CD98: An oligomeric and multifunctional protein.. Biochim Biophys Acta.

[2] Haynes BF, Hemler ME, Mann DL, Eisenbarth GS, Shelhamer J (1981). Characterization of a monoclonal antibody (4F2) that binds to human monocytes and to a subset of activated lymphocytes.. J Immunol.

[3] Fenczik CA, Sethi T, Ramos JW, Hughes PE, Ginsberg MH (1997). Complementation of dominant suppression implicates CD98 in integrin activation.. Nature.

[4] Fenczik CA, Zent R, Dellos M, Calderwood DA, Satriano J (2001). Distinct domains of CD98hc regulate integrins and amino acid transport.. J Biol Chem.

[5] Merlin D, Sitaraman S, Liu X, Eastburn K, Sun J (2001). CD98-mediated links between amino acid transport and beta 1 integrin distribution in polarized columnar epithelia.. J Biol Chem.

[6] Feral CC, Nishiya N, Fenczik CA, Stuhlmann H, Slepak M (2005). CD98hc (SLC3A2) mediates integrin signaling.. Proc Natl Acad Sci U S A.

[7] Redegeld FA, Caldwell CC, Sitkovsky MV (1999). Ecto-protein kinases: ecto-domain phosphorylation as a novel target for pharmacological manipulation?. Trends Pharmacol Sci.

[8] Imada S, Sugiyama Y, Imada M (1988). Fibronectin phosphorylation by ecto-protein kinase.. Exp Cell Res.

[9] Kubler D, Pyerin W, Burow E, Kinzel V (1983). Substrate-effected release of surface-located protein kinase from intact cells.. Proc Natl Acad Sci U S A.

[10] Litchfield DW (2003). Protein kinase CK2: structure, regulation and role in cellular decisions of life and death.. Biochem J.

[11] Meggio F, Pinna LA (2003). One-thousand-and-one substrates of protein kinase CK2?. Faseb J.

[12] Pinna LA, Meggio F (1997). Protein kinase CK2 (“casein kinase-2”) and its implication in cell division and proliferation.. Prog Cell Cycle Res.

[13] Guerra B, Issinger OG (1999). Protein kinase CK2 and its role in cellular proliferation, development and pathology.. Electrophoresis.

[14] Yan Y, Vasudevan S, Nguyen H, Bork U, Sitaraman S (2007). Extracellular interaction between hCD98 and the PDZ class II domain of hCASK in intestinal epithelia.. J Membr Biol.

[15] Skubitz KM, Ehresmann DD, Ducker TP (1991). Characterization of human neutrophil ecto-protein kinase activity released by kinase substrates.. J Immunol.

[16] Nguyen HT, Charrier-Hisamuddin L, Dalmasso G, Hiol A, Sitaraman S (2007). Association of PepT1 with lipid rafts differently modulates its transport activity in polarized and nonpolarized cells.. Am J Physiol Gastrointest Liver Physiol.

[17] Apasov SG, Smith PT, Jelonek MT, Margulies DH, Sitkovsky MV (1996). Phosphorylation of extracellular domains of T-lymphocyte surface proteins. Constitutive serine and threonine phosphorylation of the T cell antigen receptor ectodomains.. J Biol Chem.

[18] Wegener J, Keese CR, Giaever I (2000). Electric cell-substrate impedance sensing (ECIS) as a noninvasive means to monitor the kinetics of cell spreading to artificial surfaces.. Exp Cell Res.

[19] Redegeld FA, Smith P, Apasov S, Sitkovsky MV (1997). Phosphorylation of T-lymphocyte plasma membrane-associated proteins by ectoprotein kinases: implications for a possible role for ectophosphorylation in T-cell effector functions.. Biochim Biophys Acta.

[20] Kang ES, Postlethwaite A, Schaeffer S, Sawhney B (1984). Endogenous surface phosphorylation reactions and ectokinase activity in the guinea pig T lymphocyte.. Cell Immunol.

[21] Trachana V, Christophorides E, Kouzi-Koliakos K, Koliakos G (2005). Laminin-1 is phosphorylated by ecto-protein kinases of monocytes.. Int J Biochem Cell Biol.

[22] Teshima R, Saito Y, Ikebuchi H, Rajiva De Silva N, Morita Y (1997). Effect of an ectokinase inhibitor, K252b, on degranulation and Ca2+ signals of RBL-2H3 cells and human basophils.. J Immunol.

[23] Teshima R, Onose J, Saito Y, Ikebuchi H, Kitani S (1999). Casein kinase II-like ectokinase activity on RBL-2H3 cells.. Immunol Lett.

[24] Sarno S, Reddy H, Meggio F, Ruzzene M, Davies SP (2001). Selectivity of 4,5,6,7-tetrabromobenzotriazole, an ATP site-directed inhibitor of protein kinase CK2 (‘casein kinase-2’).. FEBS Lett.

[25] Zien P, Bretner M, Zastapilo K, Szyszka R, Shugar D (2003). Selectivity of 4,5,6,7-tetrabromobenzimidazole as an ATP-competitive potent inhibitor of protein kinase CK2 from various sources.. Biochem Biophys Res Commun.

[26] Redegeld F, Filippini A, Sitkovsky M (1991). Comparative studies of the cytotoxic T lymphocyte-mediated cytotoxicity and of extracellular ATP-induced cell lysis. Different requirements in extracellular Mg2+ and pH.. J Immunol.

[27] Apasov S, Koshiba M, Redegeld F, Sitkovsky MV (1995). Role of extracellular ATP and P1 and P2 classes of purinergic receptors in T-cell development and cytotoxic T lymphocyte effector functions.. Immunol Rev.

[28] Chen-Wu JL, Padmanabha R, Glover CV (1988). Isolation, sequencing, and disruption of the CKA1 gene encoding the alpha subunit of yeast casein kinase II.. Mol Cell Biol.

[29] Allende JE, Allende CC (1995). Protein kinases. 4. Protein kinase CK2: an enzyme with multiple substrates and a puzzling regulation.. Faseb J.

[30] Sarno S, Marin O, Boschetti M, Pagano MA, Meggio F (2000). Cooperative modulation of protein kinase CK2 by separate domains of its regulatory beta-subunit.. Biochemistry.

[31] Meggio F, Marin O, Pinna LA (1994). Substrate specificity of protein kinase CK2.. Cell Mol Biol Res.

[32] Litchfield DW, Arendt A, Lozeman FJ, Krebs EG, Hargrave PA (1990). Synthetic phosphopeptides are substrates for casein kinase II.. FEBS Lett.

[33] Meggio F, Perich JW, Marin O, Pinna LA (1992). The comparative efficiencies of the Ser(P)-, Thr(P)- and Tyr(P)-residues as specificity determinants for casein kinase-1.. Biochem Biophys Res Commun.

[34] Meggio F, Perich JW, Reynolds EC, Pinna LA (1991). Phosphotyrosine as a specificity determinant for casein kinase-2, a growth related Ser/Thr-specific protein kinase.. FEBS Lett.

[35] Liu X, Charrier L, Gewirtz A, Sitaraman S, Merlin D (2003). CD98 and intracellular adhesion molecule I regulate the activity of amino acid transporter LAT-2 in polarized intestinal epithelia.. J Biol Chem.

[36] Seger D, Seger R, Shaltiel S (2001). The CK2 phosphorylation of vitronectin. Promotion of cell adhesion via the alpha(v)beta 3-phosphatidylinositol 3-kinase pathway.. J Biol Chem.

[37] Stepanova V, Jerke U, Sagach V, Lindschau C, Dietz R (2002). Urokinase-dependent human vascular smooth muscle cell adhesion requires selective vitronectin phosphorylation by ectoprotein kinase CK2.. J Biol Chem.

[38] Zimina EP, Fritsch A, Schermer B, Bakulina AY, Bashkurov M (2007). Extracellular phosphorylation of collagen XVII by ecto-casein kinase 2 inhibits ectodomain shedding.. J Biol Chem.

[39] Agace WW, Higgins JM, Sadasivan B, Brenner MB, Parker CM (2000). T-lymphocyte-epithelial-cell interactions: integrin alpha(E)(CD103)beta(7), LEEP-CAM and chemokines.. Curr Opin Cell Biol.

[40] Fais S, Pallone F (1989). Ability of human colonic epithelium to express the 4F2 antigen, the common acute lymphoblastic leukemia antigen, and the transferrin receptor. Studies in inflammatory bowel disease and after in vitro exposure to different stimuli.. Gastroenterology.

[41] Schreiber S, MacDermott RP, Raedler A, Pinnau R, Bertovich MJ (1991). Increased activation of isolated intestinal lamina propria mononuclear cells in inflammatory bowel disease.. Gastroenterology.

[42] Kucharzik T, Lugering A, Yan Y, Driss A, Charrier L (2005). Activation of epithelial CD98 glycoprotein perpetuates colonic inflammation.. Lab Invest.

